# A high angiopoietin-2/angiopoietin-1 ratio is associated with a high risk of septic shock in patients with febrile neutropenia

**DOI:** 10.1186/cc12848

**Published:** 2013-08-05

**Authors:** Maiara Marx Luz Fiusa, Carolina Costa-Lima, Gleice Regina de Souza, Afonso Celso Vigorito, Francisco Jose Penteado Aranha, Irene Lorand-Metze, Joyce M Annichino-Bizzacchi, Carmino Antonio de Souza, Erich V De Paula

**Affiliations:** 1 Hematology and Hemotherapy Center, University of Campinas, Rua Carlos Chagas 480, Campinas, SP, 13083-970, Brazil; 2 Department of Internal Medicine, Faculty of Medical Sciences, University of Campinas, Rua Tessalia Vieira de Camargo 126, Campinas, SP, 13083-887, Brazil; 3 Department of Clinical Pathology, University of Campinas, Rua Tessalia Vieira de Camargo 126, Campinas, SP, 13083-887, Brazil

## Abstract

**Introduction:**

Endothelial barrier breakdown is a hallmark of septic shock, and proteins that physiologically regulate endothelial barrier integrity are emerging as promising biomarkers of septic shock development. Patients with cancer and febrile neutropenia (FN) present a higher risk of sepsis complications, such as septic shock. Nonetheless, these patients are normally excluded or under-represented in sepsis biomarker studies. The aim of our study was to validate the measurement of a panel of microvascular permeability modulators as biomarkers of septic shock development in cancer patients with chemotherapy-associated FN.

**Methods:**

This was a prospective study of diagnostic accuracy, performed in two distinct in-patient units of a university hospital. Levels of vascular endothelial growth factor A (VEGF-A), soluble fms-like tyrosine kinase-1 (sFlt-1) and angiopoietin (Ang) 1 and 2 were measured after the onset of neutropenic fever, in conditions designed to mimic the real-world use of a sepsis biomarker, based on our local practice. Patients were categorized based on the development of septic shock by 28 days as an outcome.

**Results:**

A total of 99 consecutive patients were evaluated in the study, of which 20 developed septic shock and 79 were classified as non-complicated FN. VEGF-A and sFlt-1 levels were similar between both outcome groups. In contrast, Ang-2 concentrations were increased in patients with septic shock, whereas an inverse finding was observed for Ang-1, resulting in a higher Ang-2/Ang-1 ratio in patients with septic shock (5.29, range 0.58 to 57.14) compared to non-complicated FN (1.99, range 0.06 to 64.62; *P *= 0.01). After multivariate analysis, the Ang-2/Ang-1 ratio remained an independent factor for septic shock development and 28-day mortality.

**Conclusions:**

A high Ang-2/Ang-1 ratio can predict the development of septic shock in cancer patients with febrile neutropenia.

## Introduction

Septic shock is one of the most severe complications of sepsis, frequently preceding multi-organ dysfunction syndrome and death. Cancer patients are at an increased risk of sepsis complications for several reasons, including the need for frequent invasive procedures, the effects of cancer on nutritional status and immunity, and the immunosuppressive effects of chemotherapy [1]. As life expectancy increases, so does the prevalence of age-related morbidities, such as cancer, considered one of the most important contributing factors for the increasing incidence of sepsis [2]. In this context, the discovery and validation of new biomarkers for septic shock development is regarded as a major research objective in the field [3,4]. However, the implementation of a biomarker usually involves a stepwise process, in which results from initial studies need to be confirmed in larger studies in independent populations, ideally performed in experimental environments that more closely resemble the "real-world" use of the biomarker [3,5].

In the last decade, the cellular and molecular mechanisms that regulate endothelial barrier integrity have been described [6]. In the embryo, VEGF-A, sFlt-1, angiopoietin (Ang)-1 and Ang-2 play critical roles in the physiological process of angiogenesis, during which the endothelial barrier is constantly assembled and disassembled, to allow the incorporation of new vessel sprouts. While VEGF-A and Ang-2 are involved in the destabilization of endothelial cell junctions, sFlt-1 and Ang-1 present distinct functions, stabilizing the endothelial barrier. After the initial demonstration that the mechanisms that regulate endothelial barrier formation in the embryo are also involved in the pathological disruption of the endothelial barrier in adults during sepsis [7], several studies explored the role of these proteins as biomarkers of septic shock development. Encouraging results were reported by several groups, and Ang-1 and Ang-2 emerged among the most promising endothelial-associated sepsis biomarkers [8-19].

Patients with cancer and febrile neutropenia (FN) are one of the populations with the highest risks of sepsis-related mortality [20,21]. In these patients, septic shock can evolve as a fulminant complication, despite aggressive treatment with broad-spectrum antibiotics and best supportive care. Nevertheless, these patients are usually excluded or largely underrepresented in studies about sepsis biomarkers and management. In a small exploratory study, we previously demonstrated that levels of modulators of endothelial permeability, such as VEGF-A and Ang-2 were altered in patients with FN that developed septic shock [22,23]. In the present work, we sought to validate the use of VEGF-A, sFlt-1, Ang-1 and Ang-2 levels as biomarkers of septic shock development in a larger and independent cohort of patients with cancer-associated FN, in a more clinically relevant scenario regarding the use of a sepsis biomarker.

## Material and methods

### Patients

The study was conducted at the Hematopoietic Stem Cell Transplantation and at the Hematology in-patient units of University of Campinas between March 2011 and March 2012. The study was performed in accordance with the Declaration of Helsinki, and approved by the Committee of Ethics in Research of the Faculty of Medical Sciences, University of Campinas, Campinas, SP, Brazil. Written informed consent was obtained from all patients prior to any study procedure. Inclusion criteria were: (1) fever (T ≥38°C) over a one-hour observation period, (2) chemotherapy-induced severe neutropenia, characterized by an absolute neutrophil count <0.5 × 10^9^/L at the time of fever onset, and (3) persistence of severe neutropenia until the time of blood sample collection. Demographic and clinical data were obtained from the medical records.

### Sepsis definitions and clinical scores

In accordance with FN management protocols, an infectious etiology was assumed for all patients with post-chemotherapy neutropenia with new onset of fever [24]. Blood and urine cultures were immediately obtained and broad spectrum antibiotics initiated, along with the infusion of 1.5 L of intravenous saline solution over each 24-hour period. All patients underwent chest radiography. Other imaging studies were performed when judged necessary by the attending physician. Sepsis was defined by the presence of two or more of the following: (1) temperature >38°C, (2) heart rate >90 beats/minute, (3) respiratory rate >20 breaths/minute or PaCO2 <32 mmHg, and a microbiologically proven or clinically evident source of infection. A diagnosis of septic shock was established when sepsis-induced hypotension (systolic arterial pressure <90 mmHg or dropping >40 mmHg from baseline) persisted despite adequate volume resuscitation. Volume resuscitation consisted of 30 ml/Kg of crystalloids (usually 2 L) in a bolus fashion over 60 minutes, targeted to a mean arterial pressure above 65 mmHg. Failure to achieve this target within one hour established the diagnosis of septic shock [25]. Severity of illness was assessed by calculating the Sequential Organ Failure Assessment (SOFA) score [26], and FN risk stratification was performed by calculation of the Multinational Association for Supportive Care In Cancer (MASCC) score [27]. Both scores were calculated at the time of sample collection.

### Study design

This was a prospective non-interventional study performed according to the STARD (Standard for the Reporting of Diagnostic Accuracy Study) guidelines [28]. All in-patients from the hematology or from the bone marrow transplantation units with new onset FN that met the inclusion criteria were invited to participate. The study was deliberately designed to mimic the conditions in which a sepsis biomarker would be used. Patients were enrolled in the study within 24 hours from admission for FN treatment, and samples were drawn with the next routine sample collection requested for them (usually in the morning after enrollment). Furthermore, samples were collected and initially processed by the hospital laboratory staff using the same operational procedures used for other patients. Finally, neither the prior use of antibiotics, nor the time elapsed from fever onset until blood draws were considered exclusion criteria for the study, as long as the patients remained with severe neutropenia at the time of sample collection. As per local protocol, the first routine sample collection requested for all admitted FN patients includes a complete blood count (in which the persistence of severe neutropenia was confirmed) and C-reactive protein (CRP) measurement.

Serum levels of VEGF-A, sFlt-1, Ang-1 and Ang-2 were measured by an individual blinded to patient outcomes, in duplicate, using commercial enzyme linked immunosorbent assay (ELISA) kits (Quantikine, R&D Systems, Minneapolis, MN, USA) according to the manufacturer's instructions. Samples were centrifuged at 1,000 g (4°C, 15 minutes) and serum was stored at -80°C until analysis. The sensitivities of the assays for VEGF-A, sFlt-1, Ang-1 and Ang-2 were 9 pg/ml, 3.5 pg/ml, 3.4 pg/ml and 8.3 pg/ml, respectively. The intra- and interassay coefficients of variation of all tests were within the range informed by the manufacturer (range 2.4% to 10.4%). Based upon a power analysis, and considering an expected rate of septic shock of 25% (from our previous study), a sample size of 100 patients would produce at least 80% power at the 0.05 level of significance to detect an effect-size of 1 SD in all four assays between study groups.

### Statistical analysis

The primary endpoint of the study was development of septic shock at any time point before the resolution of FN or at a maximum of 28 days after enrollment (in cases of prolonged neutropenia). Based on this outcome, patients were divided in two groups for the analysis: (i) non-complicated FN or (ii) septic shock. Differences in continuous variables between patients from each subgroup were analyzed using the Mann-Whitney test. Categorical variables were compared using the Fisher's exact test. Data are expressed as median and range unless otherwise stated. Correlation (Spearman's rank correlation) analysis was performed between sepsis severity scores and biomarker levels. Receiver operator characteristics (ROC) procedures were used to identify optimal cut-off values of biomarker concentrations to differentiate distinct outcome groups. To evaluate the effect of the Ang-2/Ang-1 ratio as a continuous variable on the risk of septic shock development and sepsis-related mortality, a Cox proportional-hazards model was used assuming a graded relation between the ratio and either outcome. To evaluate whether there was a non-linear association between the risk of septic shock and the Ang-2/Ang-1 ratio, a binary logistic regression model was used, in which the ratio was dichotomized by the median and by an optimal cut-off. All multivariate analysis models were adjusted for covariates with a *P-*value <0.10 in univariate analysis. Variables from the SOFA score were evaluated individually, to avoid the influence of chemotherapy-induced thrombocytopenia on the assessment of organ failure. Survival curves were estimated using the Kaplan-Meier method. A *P-*value less than or equal to 0.05 was considered statistically significant. All statistical analysis were performed with the SPSS package v. 17.2 (SPSS, Inc., Chicago, IL, USA) and the GraphPad Prism Software (GraphPad Prism Software, Inc., San Diego, CA, USA).

## Results

### Patients' characteristics

**Figure 1:**
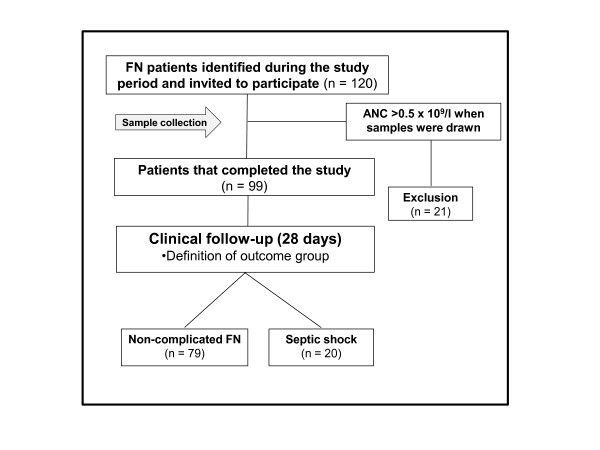
**Study flowchart**.

One-hundred twenty patients with FN were identified in both in-patient units during the study period. All patients agreed to participate, but only the 99 patients that met all inclusion criteria were maintained in the study. Twenty-one patients were excluded because the absolute neutrophil count had increased to above 0.5 × 10^9^/L at the time of sample collection (Figure 1). Of note, none of these 21 patients developed septic shock. Of the 99 episodes of FN, 20 (19.8%) evolved to septic shock, and 17 (16.8%) were fatal. All deaths were attributed to complications of septic shock, and occurred after a median of 13 days from sample collection (range 3 to 28). Seventy-eight FN episodes (78.8%) were classified as high-risk FN, based on a MASCC score ≤21 [29]. There were no significant clinical and demographic differences between patients with non-complicated FN and septic shock with respect to age, diagnosis, MASCC score, CRP, neutrophil and platelet counts, nor any of the components of the SOFA score. A higher SOFA score in patients that developed septic shock was the only significant difference observed at baseline between the two outcome groups (Table 1). The median time to septic shock development after sample collection was three days (range 1 to 17, interquartile range 4.5). In total, five patients developed septic shock more than seven days after fever onset. In four of them, septic shock was associated with probable or proven invasive fungal infections. In addition, three patients developed septic shock between enrollment and sample collection, within 12 hours from blood draw.

**Table 1:** Patient characteristics

	Non-complicated FN (n = 79)	Septic shock (n = 20)	*P*
**Gender **(male: female)	44:35	12:8	NS**
**Age **(median, range)	49 (13 to 78)	52 (21 to 78)	NS**
**Diagnostic **NS**			
Acute leukemia	30	10	
Other hematolgical malignancies	49	10	
**Comorbidities **(present:absent)	29:40	8:12	NS**
**Treatment **NS**			
Intensive CTx/autologus HSCT	62	15	
Allogeneic HSCT	17	5	
**Neutrophils**/µl (median, range)	80 (0 to 500)	0 (0 to 500)	NS*
**Platelets x10³/µl **(median, range)	**21,000** (4,000 to 215,000)	**14,500** (3,000 to 39,000)	NS*
Agent isolation in bloodstream*** (yes:no)	27:52	7:13	NS**
**C reactive protein mg/dl **(median, range)	11.20 (0.55 to 58.30)	12.7 (6.2 to 40.90)	NS*
**SOFA score **(median, range)	4 (0 to 8)	4 (2 to 14)	*P *<0,05*
**MASCC score **(median, range)	19 (12 to 23)	18 (12 to 21)	NS*
**Time to sample collection **hours (median, range)		48 (3 to 120)	
**Time to septic shock **days (median, range)		3 (1 to 17)	

CTx, chemotherapy; HSCT, hematopoietic stem cell transplantation; MASCC, Multinational Association for Supportive Care in Cancer; SOFA, Sequential Organ Failure Assessment; * Mann-Whitney test; ** Fisher's exact test; *** Gram-negative: Acinetobacter baumannii, Enterobacter cloacae, Escherichia coli, Klebsiella pneumoniae, Pseudomonas aeruginosa, Stenotrophomonas maltophilia. Gram-positive: Staphylococcus aureus, Staphylococcus epidermidis, Streptococcus viridans, Staphylococcus warneri, Staphylococcus haemolyticus. Fungi: Aspergillus e Fusarium.

### Serum levels endothelial permeability modulators according to the development of septic shock

**Figure 2:**
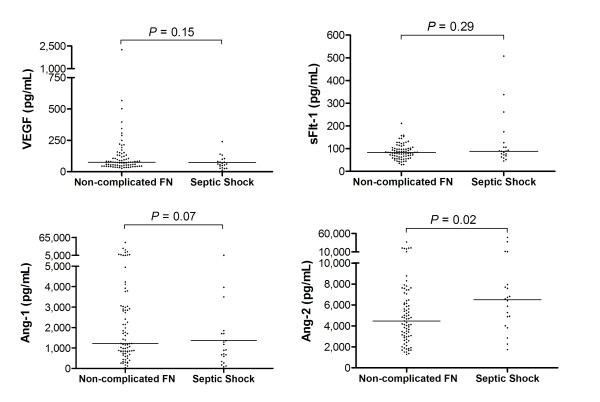
**Vascular permeability-related biomarkers in FN patients**. Serum concentrations of soluble fms-like tyrosine kinase-1 (sFlt-1), vascular endothelial growth factor-A (VEGF-A) and angiopoietin (Ang) -1 and Ang-2 in patients with non-complicated febrile neutropenia (FN) (n = 79) or septic shock (n = 20). Horizontal bars represent median values; Mann-Whitney test.

No statistically significant difference could be detected between VEGF-A and sFlt-1 levels in patients with non-complicated FN compared to patients that developed septic shock (Figure 2). Serum concentrations of Ang-1, an endothelial barrier-stabilizing factor, were higher in patients with non-complicated FN (1,220.0 pg/ml, range 32.5 to 47,924.0 pg/ml) than in patients with septic shock (898.8 pg/ml, range 77.9 to 5,420.0 pg/ml), although this difference did not reach statistical significance (Mann-Whitney test, *P *= 0.07) (Figure 2). Levels of Ang-2, an endothelial barrier-destabilizing factor, were higher in patients that developed septic shock (6,494.0 pg/ml, range 1,730.0 to 49,611.0 pg/ml) compared to patients with non-complicated sepsis (4,467.0 pg/ml, range 1,289.0 to 37,318.0 pg/ml; Mann-Whitney test: *P *= 0.02) (Figure 2).

In addition to these findings, serum levels of Ang-1 presented a negative correlation with the SOFA score (Rs = -0.36; *P *= 0.0002) and serum levels of Ang-2 a positive correlation with CRP (Rs = 0.3; *P *= 0.002). No correlation could be observed between platelet counts and Ang-1 (Rs = 0.1; *P *= 0.34) or Ang-2 (Rs = 0.1; *P *= 0.34) levels. Interestingly, when only patients with a platelet count below the median were evaluated, a significant correlation between Ang-1 and platelet count could be observed (Rs = 0.36; *P *= 0.007).

### The Ang-2/Ang-1 ratio is increased in patients with FN neutropenia that will develop septic shock compared to patients with non-complicated FN

**Figure 3:**
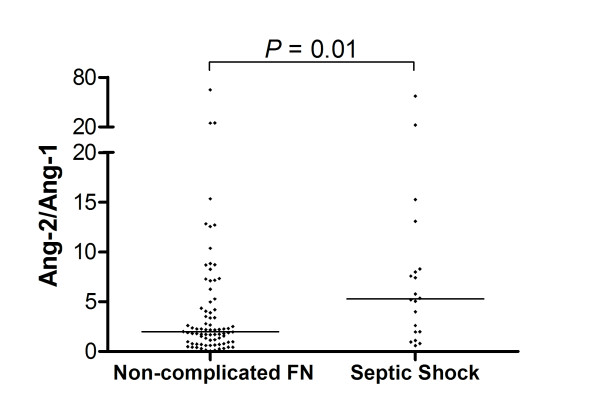
**Ang-2/Ang-1 ratio in FN patients**. Angiopoietin- (Ang)-2/Ang-1 ratio in patients with non-complicated febrile neutropenia (FN) (n = 79) or septic shock (n = 20). Horizontal bars represent median values; Mann-Whitney test.

Because of their antagonistic roles for endothelial barrier integrity, the relative concentrations of Ang-1 and Ang-2, expressed as the Ang-2/Ang1 ratio, has been considered a more relevant sepsis biomarker than isolated levels of each factor. In fact, the Ang-2/Ang-1 ratio was much higher in the septic shock outcome group (5.3, range 0.6 to 57.1) compared to patients with non-complicated FN (1.9, range 0.1 to 64.6; Mann-Whitney test: *P *= 0.01) (Figure 3).

Estimation of the diagnostic accuracy of the Ang-2/Ang-1 ratio for the development of septic shock yielded an area under the ROC curve of 0.68 (95%CI = 0.55 to 0.81; *P *= 0.01). An Ang-2/Ang-1 ratio of 5.0 was the optimal cut-off value identified by the ROC procedure, with a sensitivity of 60% (95% CI = 36.1% to 80.9%) and a specificity of 77.2% (95% CI = 66.4% to 85.9%) for the development of septic shock. The positive and negative predictive values were 47% and 85%, respectively. Of note, the estimated diagnostic accuracy of the Ang-2/Ang-1 ratio was higher than that obtained with either Ang-1 or Ang-2 levels alone, as well as that of CRP, SOFA or the MASCC scores (Table 2).

**Table 2:** Diagnostic accuracy for septic shock development

	AUC*	95% Confidence interval	*P*
**SOFA**	0.65	0.52 to 0.79	0.03
**MASCC**	0.59	0.45 to 0.74	0.19
**C reactive protein**	0.59	0.46 to 0.73	0.19
**VEGF-A**	0.60	0.46 to 0.74	0.15
**SFlt-1**	0.57	0.43 to 0.72	0.29
**Ang-1**	0.63	0.49 to 0.76	0.08
**Ang-2**	0.66	0.53 to 0.79	0.03
**Ang-2/Ang-1 ratio**	0.68	0.55 to 0.81	0.01

*AUC, area under the ROC curve

### An Ang-2/Ang-1 ratio above 5.0 is an independent factor for the development of septic shock and sepsis-related mortality in patients with FN

When the Ang-2/Ang-1 was analyzed as a continuous variable in a Cox proportional-hazards model, the relative risk of septic shock was 1.06 (95% CI 1.02 to 1.10; *P *= 0.004), even after adjustment for neutrophil and platelets counts. Other variables, including age, sex, baseline diagnosis, treatment type and CRP, among others, were not associated with the risk of septic shock development in our population. This first analysis assumed that the association between the Ang-2/Ang-1 ratio and the risk of septic shock development presented a graded relation. We next evaluated whether there was a threshold level of the Ang-2/Ang-1 ratio above which the risk of septic shock would increase in a non-linear fashion. We dichotomized levels of Ang-2/Ang-1 into median and cut-off values (estimated by the ROC procedure), and calculated the relative risks of septic shock development. The risk of septic shock was almost five times greater among patients with Ang-2/Ang-1 ratio exceeding the cut-off, even after adjustment for neutrophil and platelet counts (covariates with a *P *<0.1 in univariate analysis) (Table 3).

**Table 3:** Relative risk of septic shock development

	Chi-square test		Multivariate analysis*	
**Ang-2/Ang-1 (n = 99)**	**Odds ratio** **(Cl 95%)**	***P***	**Odds ratio** **(Cl 95%)**	

*>Median*	2.90 (1.0 to 8.4)	0.04	2.99 (1.02 to 8.42)	0.045
*>Cutoff*	5.50 (1.9 to 16)	0.0007	5.47 (1.93 to 15.53)	0.001

* Binary logistic regression

### Ang-2/Ang-1 ratio and 28-day sepsis-related mortality in patients with FN

**Figure 4:**
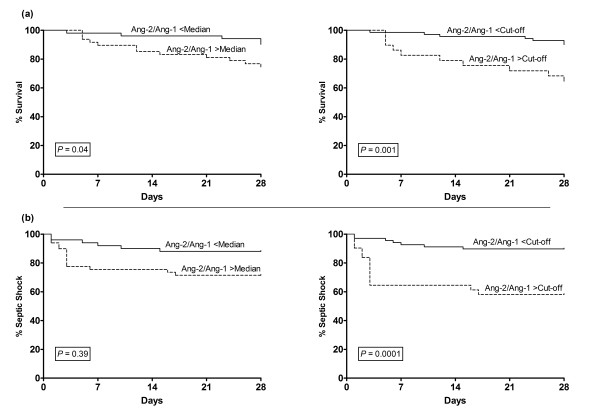
**Twenty-eight-day survival and septic shock development in high-risk FN patients**. Kaplan-Meier estimates of 28-day (**a**) survival and (**b**) septic shock development of patients with febrile neutropenia (FN) according to angiopoietin (Ang)-2/Ang-1 ratio. The ratio was dichotomized by the median Ang-2/Ang-1 ratio (2.2) in all patients (right) or by an optimal cut-off level of 5.0 (left), defined by a receiver operator characteristic (ROC) analysis.

The relative risk of sepsis-related mortality for patients with Ang-2/Ang-1 levels above the 5.0 cut-off was 5.8 (95% CI 1.96 to 17.59; *P *= 0.001) and 2.74 (95% confidence interval, 1.054 to 7.160; *P *= 0.03) for Ang-2/Ang-1 levels above the median. Using a Cox proportional-hazards model, adjusting for neutrophil and platelet count, the mortality risk was 4.20 (95% CI 1.60 to 11.05; *P *= 0.004) for patients with an Ang-2/Ang-1 ratio above 5.0, and 2.73 (95% CI 0.97 to 7.75; *P *= 0.059) for patients with an Ang-2/Ang-1 ratio above the median. The Kaplan-Meier curves showed clear divergence of survival estimates between patients with Ang-2/Ang-1 ratios above or below the median or cut-off values (Figure 4).

## Discussion

Among the several factors that contribute to a higher risk of sepsis complications in patients with cancer, chemotherapy-associated neutropenia is probably the single-most important one [29]. Although the risk is higher in patients with severe and long-lasting neutropenia, risk stratification tools are limited, and the majority of patients end up treated aggressively with hospitalization and broad-spectrum antibiotics [29]. The most accepted risk stratification tool for cancer-related FN is the MASCC score [27]. However, this score offers limited information for patients categorized as high-risk (MASCC ≤21), which encompasses most patients submitted to more intensive myelotoxic chemotherapy protocols. Accordingly, management of patients with cancer-related FN remains a challenging situation, for which the availability of informative biomarkers would be welcome.

In the last decade, mechanisms by which the endothelial barrier is established in the embryo have been described in detail [6]. As they were being described, some authors demonstrated that the same mechanisms were involved in the pathological disruption of the endothelial barrier observed during sepsis [7]. Soon after that, encouraging results were published about the use of VEGF-A, sFlt-1, Ang-1 and Ang-2 as biomarkers of septic shock development. In general, these studies demonstrated a consistent pattern in patients with septic shock, characterized by increased levels of proteins that loosen endothelial cell junctions during vessel formation, such VEGF-A and Ang-2, and decreased levels of barrier stabilizing proteins, such as Ang-1. Indeed, imbalances in Ang-1/Ang-2 ratio, expressed by a high Ang-2/Ang-1 ratio, recently emerged as one of the most promising biomarkers for septic shock development in several populations [11,16,30]. However, because patients with cancer were excluded or underrepresented in these studies, it is not known whether these results may be applied to patients with cancer.

In a preliminary study, we demonstrated that VEGF-A, sFlt-1, Ang-1 and Ang-2 were associated with sepsis severity in patients with FN [22,23]. So, the objective of the present study was to validate the use of these biomarkers in a larger and independent cohort of patients with cancer and chemotherapy-associated FN, in experimental conditions more closely mimicking the scenario in which a sepsis biomarker would be ordered. This was achieved by: (1) sequential enrollment of patients; (2) limitation of exclusion criteria; (3) sample collection and initial processing performed by hospital staff; and (4) sampling time mimicking the local use of a biomarker (CRP) already used for patients with FN [14].

Not infrequently, results obtained in well-designed studies of diagnostic accuracy are not reproduced in similar independent populations [31], due to differences in time of sample collection and even in sample type and manipulation. In fact, we were not able to confirm previous reports of VEGF-A or sFlt-1 as informative biomarkers of septic shock development in patients with FN [23,32]. We speculate that these discrepancies are associated with differences in the time-point of sample collection, and with the potential influence of severe neutropenia and thrombocytopenia in the variation of VEGF-A, as neutrophils and platelets are major sources of these mediator [33]. In addition, we cannot rule out the possibility that differences in baseline levels of VEGF-A between different types of malignancies [34] could have also played a role. Whatever the reasons for these discrepancies, they suggest that assays for VEGF-A and sFlt-1 possibly lack the robustness required for a sepsis biomarker to overcome the challenge of "real world" conditions, based on our local-practice, in the context of FN.

On the other hand, the main finding of our study was the confirmation that the Ang-2/Ang-1 ratio is independently associated with septic shock development in patients with cancer and high-risk FN, for whom other risk stratification tools provide very limited information. Although it should be noted that the relatively low sensitivity and specificity of the Ang-2/Ang-1 ratio do not qualify it as a biomarker that could influence decision-making by itself, it should also be pointed out that it presented a higher estimated diagnostic accuracy than CRP, Ang-1, Ang2 or the SOFA and MASCC scores, with the latter being the most widely used risk stratification tool for this population. In addition, the sensitivity and specificity of the Ang-2/Ang-1 ratio was similar to that of procalcitonin for the diagnosis of sepsis in critically ill patients [35]. Finally, our findings are important not only because a biomarker with a negative predictive value of 85% deserves to be evaluated as part of decision-making algorithms that incorporate clinical and laboratory markers in patients with FN, but also because they provide additional insights into the pathogenesis of septic shock in this particular population.

The use of the Ang-2/Ang-1 ratio rather than Ang-2 as a biomarker has been a matter of recent debate [36]. As opposed to Ang-2, which is exclusively stored in endothelial cells, Ang-1 is also expressed in platelets [37], so that *ex vivo *platelet activation can result in falsely elevated Ang-1 levels, limiting the accuracy of the Ang-2/Ang-1 ratio. Accordingly, the effect of pre-analytical variables, such as sample type and manipulation, have been cited as possible explanations for discrepant results of Ang-1 levels as a sepsis biomarker [36]. On the other hand, it is tempting to speculate that "true" imbalances in Ang-1 and Ang-2 levels do represent a biologically relevant determinant of endothelial barrier integrity, as suggested by several lines of evidence [38]. It is not known whether the effect of *ex vivo *platelet activation on Ang-1 levels is influenced by sample platelet count. However, our data showing a significant correlation between platelet count and Ang-1 levels only in patients with severe thrombocytopenia, but not in patients with higher and more variable platelet counts, suggest that severe hypoplastic thrombocytopenia could attenuate the influence of the pre-analytical variables that normally limit the determination of "true" Ang-1 levels. Therefore, although we acknowledge the technical limitations of Ang-1 determination, we speculate that more reliable Ang-1 levels can be obtained in patients with chemotherapy-induced severe thrombocytopenia.

One of the main objectives of our study was to determine whether Ang-1 and Ang-2 measurements would remain as relevant sepsis biomarkers in a population with different characteristics than those from studies of non-neutropenic patients. Neutrophils are critical components of the innate immune response, releasing cytokines and chemokines, engulfing pathogens and contributing to microbial killing by a range of peptides and by neutrophil extracellular trap formation [39]. It was interesting to note that the optimal cut-off of the Ang-2/Ang-1 ratio associated with septic shock in our population was very similar to the mean peak Ang-2/Ang-1 ratio observed in non-survivors in a study with non-neutropenic sepsis patients (5.0 vs 5.4, respectively) [11]. From a clinical standpoint, a significant difference between our population and non-neutropenic patients is the earlier collection of blood samples in the course of infection, when clinical signs of sepsis were mild or absent (as illustrated by the lower SOFA score in our patients). The reason for this characteristic lies in the fact that patients with FN are instructed to look for medical care immediately after fever onset, allowing earlier admission. In fact, we believe that the relatively lower Ang-2 levels in our patients are another consequence of this earlier evaluation. Of note, we have previously shown a five-fold increase in Ang-2 levels during the first 48 hours of FN in patients that evolved to septic shock [22]. Another important difference in septic shock between neutropenic and non-neutropenic patients is the high frequency of invasive fungal infections (IFI) in the former group, often associated with a protracted course of fever, and a longer time to septic shock. In fact, although most of our patients evolved to septic shock within a week from fever onset, four of the five patients in whom septic shock developed from 7 to 17 days presented probable or proven IFI. Together, the fact that a similar value of the Ang-2/Ang-1 ratio was associated with worse outcomes in these two different populations strengthens the concept that imbalances in Ang-2 and Ang-1 concentrations are indeed a relevant biomarker of septic shock development. Moreover, the fact that imbalances in this pathway remain relevant for the pathogenesis of septic shock in patients with severe neutropenia indicates the independence of the Tie/Angiopoietin pathway from neutrophil function during sepsis. Indeed, this concept is reinforced by a series of other recent studies showing that manipulation of angiopoietin pathways can improve sepsis outcomes in different animal models [15,40,41].

This study has several limitations. First, because of a higher than expected coefficient of variation of VEGF-A levels in our patients (CV% of 193%), and with only 20 patients with septic shock, our study had power to detect only extreme differences of VEGF-A levels between the outcome groups. Second, we did not evaluate baseline Ang-2 levels. So, it is conceivable that differences in Ang-2 levels present before chemotherapy, which have been shown to be associated with disease-free survival in high-risk myeloid malignancies [42], might have influenced our results. Arguing against this possibility is the fact that we and others have not found significant differences in pre-chemotherapy Ang-2 levels between different disease groups, even in cancers with significant angiogenic activity, such as multiple myeloma [22,42]. A third limitation refers to the importance of the SOFA score as a readout of systemic organ failure in our population, since thrombocytopenia was the only category in which 61% of our patients scored, most of them with high scores. Since thrombocytopenia in our patients is simply a transient effect of chemotherapy, it is fair to assume that the diagnostic accuracy of this score is more limited in our population. We tried to minimize this limitation by avoiding the use of the score (mainly driven by thrombocytopenia) in our multivariate analysis, in which platelet count had already been used for adjustment. Fourth, we did not evaluate other outcomes that have also been associated with alterations of the Ang/Tie pathway, such as acute lung injury [43], so that our results can only be applicable to the context of septic shock development. Fifth, we did not control the effect of fluid management, which has been shown to influence Ang-2 levels [8], so that it is not possible to exclude the idea that more aggressive fluid therapy could have resulted in higher Ang-2 levels in some patients. However, extreme values of Ang-2 were observed in both outcome groups, a consistent finding in studies with this biomarker. Furthermore, fluid management before sample collection was fairly standardized in our study. Last, because three of our patients developed septic shock between enrollment and sample collection, our study does not support the use of the Ang-2/Ang-1 ratio as a predictive tool, but rather as a biomarker of septic shock development in a high-risk population.

Finally, we believe that an additional use of the Ang-2/Ang-1 ratio in sepsis in the future could be the identification of septic patient subgroups, in which disruption of the endothelial barrier could play a more prominent pathogenic role. In theory, the identification of these more "leaky" patients could rationally guide the use of new therapeutic strategies targeting pathways involved in endothelial barrier regulation [13,44]. As targeted-therapies directed to different pathogenic mechanisms of sepsis begin to reach the phase of human clinical trials, the availability of biomarkers capable of identifying discrete patient subgroups among the highly heterogeneous population of patients with sepsis, could well be the difference between failure and success of new therapies for sepsis [4].

## Conclusions

In conclusion, our study confirms that the measurement of the Ang-2/Ang-1 ratio is a biomarker of septic shock development in patients with cancer and chemotherapy-related FN.

## Key messages

• Ang-1 and And-2 are key regulators of endothelial barrier integrity, and seem to be involved in the pathological disruption of endothelial barrier during sepsis.

• Levels of Ang-1 and Ang-2 have been shown to be promising biomarkers of septic shock development in several populations, but not in cancer patients.

• A high Ang-2/Ang-2 ratio is independently associated with septic shock development in patients with cancer and chemotherapy-associated febrile neutropenia.

## Abbreviations

Ang: angiopoietin; CRP: C-reactive protein; CV%: coefficient of variation; ELISA: enzyme linked immunosorbent assays; FN: febrile neutropenia; IFI: invasive fungal infections; IRB: institutional review board; MASCC: Multinational Association for Supportive Care In Cancer; ROC: receiver operator characteristics; sFlt-1: soluble fms-like tyrosine kinase-1; SOFA: Sequential Organ Failure Assessment; STARD: Standards for the Reporting of Diagnostic Accuracy Studies; VEGF: vascular endothelial growth factor

## Competing interests

The authors declare that they have no competing interests.

## Authors' contributions

MMLF enrolled patients, recorded clinical data, performed laboratory analysis and drafted the manuscript. CC, GRS, ACV and FJPA enrolled patients and contributed to manuscript production. IL, JMA and CADS contributed to study design, data analysis and reviewed the manuscript. EVDP designed the study, analyzed the data and contributed to manuscript production. All authors read and approved the final manuscript for publication.
